# Electrical Properties of Schottky Devices from HfO_2_ and ZnO/HfO_2_ Thin Films: Morphological, Structural, and Optical Investigations

**DOI:** 10.1021/acsomega.4c06878

**Published:** 2025-02-12

**Authors:** Ayten Seçkin, Haluk Koralay

**Affiliations:** † Basic and Engineering Sciences Central Laboratory Application and Research Center (GUTMAM), Gazi University, Ankara 06560, Turkiye; ‡ Department of Physics, Faculty of Science, Gazi University, Ankara 06560, Turkiye

## Abstract

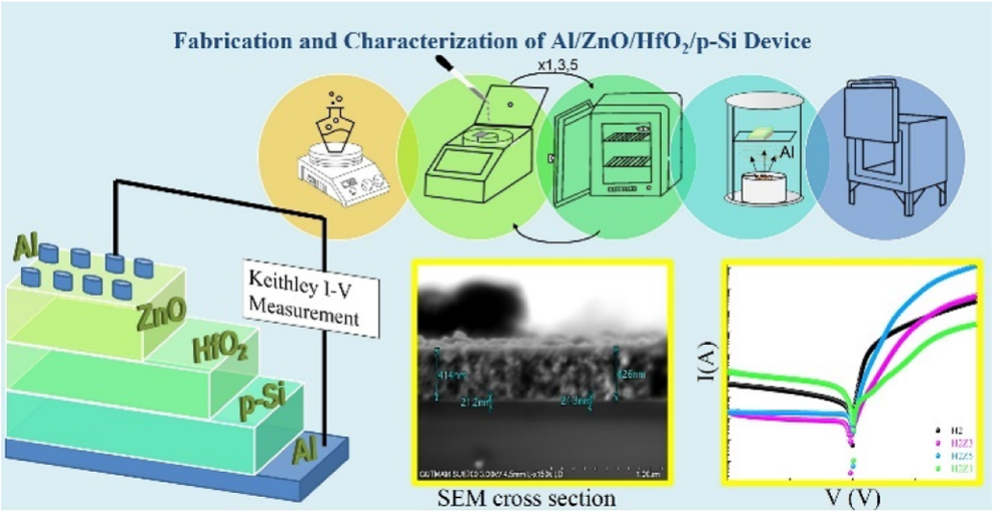

The structural and electronic properties of thin films are crucial for the performance of heterojunction diodes, which are key components in modern electronic devices. Optimizing these properties through advanced materials and fabrication techniques is an area of significant research, particularly in reducing leakage currents and enhancing device reliability. This study investigates the structural characteristics of a monolayer HfO_2_ and a double layer ZnO/HfO_2_ interface layer. This research specifically examines the impact of an HfO_2_ interface layer on the current transport properties of *n*-ZnO/p-Si heterojunction diodes, because understanding the current transport mechanisms in ZnO and HfO_2_ thin films, especially in relation to reducing defects and leakage currents, presents a challenge. Current–voltage characterization reveals that these diodes, grown by sol–gel spin coating. exhibit current transport behavior consistent with tunneling, with exponential trap distributions contributing under high voltage bias conditions. A thermionic emission (TE) mechanism is observed at low voltages (*V* < 0.4 V), followed by space-charge limited conduction (SCLC) at medium voltages (*V* < 0.5 V), and a trap charge limited current (TCLC) mechanism at high voltages (*V* > 1 V) in the dark forward current–voltage characteristics. The incorporation of the *n*-ZnO/HfO_2_/p-Si structure significantly reduces leakage currents associated with defects. These findings advance the understanding of ZnO/HfO_2_-based heterojunction diodes and pave the way for their potential application in more efficient electronic devices. The X-ray diffraction (XRD) spectra have revealed that all films crystallize in the hexagonal wurtzite structure. Structural parameters such as crystallite size, dislocation density, and microstrain in the crystal structure have been calculated. The coating thicknesses and elemental distributions of thin-film samples were determined from Field emission scanning electron microscopy (FE-SEM) images obtained from both surface and cross-sectional views. The energy band gaps of HfO_2_ and ZnO/HfO_2_ thin films were determined using absorption measurements obtained with a ultraviolet (UV) spectrophotometer. The surface roughness and topography information on thin-film samples were determined from Atomic force microscopy (AFM).

## 1 Introduction

Metal–semiconductor (MS) junctions are vital components in optoelectronic and electrical devices, playing a critical role in the efficiency and performance of semiconductor technology, and their efficiency is crucial for the industry, broadly speaking. The primary technological challenge for MS-type Schottky diodes is to use materials with excellent dielectric characteristics as interfacial layers to increase their performance and reduce their prices.^1,2^

There are several ways to improve the MS interface. One of these is to deposit an insulating or metal oxide interfacial layer between metal and semiconductor, as oxide interfacial layers are known to significantly influence the stability, reliability, and efficiency of these structures.^3^

When an oxide or an insulating layer is deposited between the metal and semiconductors, MS structures are called metal insulator semiconductor (MIS) or metal oxide semiconductor (MOS) structures. The utilization of the oxide and insulator layer among MS determines the stability, reliability, and efficiency of these structures and also prevent diffusion among MS and adjusts the charge transitions.^3^ Furthermore, the conditions of surface preparation and the creation of these interface layers impact the electronic characteristics of the MOS or MIS structures.

In current technology, using a SiO_2_ insulator layer between the metal and semiconductor to passivate dangling bonds at the semiconductor surface and reduce leakage current is no longer sufficient.^4−6^ As MOS-based devices continue to scale down, the thickness of SiO_2_ (d(SiO_2_)) is expected to decrease to the sub-2 nm range. However, challenges such as reliability issues, low drive current, and high leakage (tunneling) current make such a reduction in d(SiO_2_) problematic.^7^ The most significant advancement in this area has been the replacement of traditional SiO_2_-based gate insulators with higher permittivity (high-*k*) oxides. These oxides must meet several stringent criteria, including a low interface trap density (<10^11^ cm^–2^), thermodynamic stability in contact with silicon, and a band gap greater than 5 eV, to ensure sufficiently large band offsets at the interface with silicon.^8^ One approach to overcoming these challenges is to use insulators with a dielectric permittivity (ϵ) higher than that of SiO_2_ (ϵ ≈ 3.9).^9^ Alternatives such as Si_3_N_4_ (ϵ ∼ 7.5),^9^ Al_2_O_3_ (ϵ ∼ 8.5),^10^ ZrO2 (ϵ ∼ 20),^11^ and HfO_2_ (ϵ ∼ 22)^12^ have been extensively studied.^13^

Among these, HfO_2_ stands out due to its much higher dielectric constant (∼25) and favorable electrical properties, including thermal and chemical stability, high breakdown voltage, and low leakage current.^14−17^ Despite its advantages, HfO_2_ can produce a high Schottky barrier when in contact with high work function metals. Recent research has extensively investigated MOS structures incorporating HfO_2_ as an interface layer.^18−22^

In parallel, Zinc oxide (ZnO) has attracted considerable attention due to its substantial exciton binding energy and wide direct band gap.^23,24^ ZnO’s low cost, good chemical stability, biocompatibility, and high electron mobility make it suitable for various optoelectronic devices, including ultraviolet (UV) detectors,^25,26^ thin-film transistors (TFTs),^27,28^ light-emitting diodes,^29,30^ sensors,^31^ photovoltaic,^32^ Resistive random-access memory (ReRAM).^33−36^ Numerous studies have reported on ZnO/p-Si-based Schottky barrier diodes.^37−42^

Given the current interest in ZnO and HfO_2_, understanding the combined effects of these materials in a p-Si MIS/MOS structure is essential. In this study, we prepared four samples of heterojunction diodes, each featuring either a single layer of HfO_2_ or multilayer of ZnO/HfO_2_, using the sol–gel spin-coating method. These samples were designed to investigate the structural, optical, and electrical properties of the diodes, particularly focusing on the impact of ZnO thin films of varying thicknesses on the HfO_2_/p-Si structure. In addition to examining the conduction mechanisms through current–voltage (*I*–*V*) measurements, we also analyzed key parameters such as ideality factor, barrier height, series resistance, and interface state density.

To address this research question, we prepared four samples of heterojunction diodes, each featuring either a single layer of HfO_2_ or a bilayer of ZnO/HfO_2_, using the sol–gel spin-coating method. These samples were designed to investigate the structural, optical, and electrical properties of the diodes, particularly focusing on the impact of ZnO thin films of varying thicknesses on the HfO_2_/p-Si structure. In addition to examining the conduction mechanisms through current–voltage (*I*–*V*) measurements, we also analyzed key parameters such as ideality factor, barrier height, series resistance, and interface state density. Furthermore, the morphological, structural, and optical properties of the HfO_2_ and ZnO/HfO_2_ thin films were thoroughly evaluated through detailed analyses using X-ray diffraction (XRD), field emission scanning electron microscopy (FE-SEM), energy dispersive spectroscopy (EDS), atomic force microscopy (AFM), and UV spectroscopy.

Our study provides the first comprehensive analysis of the electrical properties of ZnO thin films with varying thicknesses on HfO_2_/p-Si structures. The results reveal significant insights into the conduction mechanisms, highlighting the potential of combining HfO_2_ and ZnO layers to enhance the performance of p-Si MIS/MOS structures. These findings contribute to a deeper understanding of the interface characteristics and open up new possibilities for optimizing the design and fabrication of high-performance electronic devices. By demonstrating the advantages of HfO_2_ and ZnO in reducing leakage currents and improving overall device efficiency, this research advances the field of semiconductor technology and paves the way for future innovations in electronic and optoelectronic applications.

## 2 Experimental Section

### 2.1 Fabrication of HfO_2_ and HfO_2_/ZnO Thin Films

A p-type silicon wafer with a 100 orientation, a resistivity of 10^–20^ Ω·cm, and a 2-in. diameter was selected and then divided into four equal parts. To clean all the Si wafer pieces, chloroform, acetone, ethanol, and deionized water were used in this order with each solution being applied for 5 min. Subsequently, Radio Corporation of America (RCA)^43^ and hydrofluoric acid (HF) cleaning methods were applied, respectively. To prepare the HfO_2_ sol, HfCl_4_ was dissolved in ethanol, deionized water, and acetic acid, and then monoethanolamine was added as a stabilizer. To prepare HfO_2_ sol, this solution was mixed for 3 h at room temperature. The solution was filtered and then deposited on all Si wafer substrates at 500 rpm for 15 s by spin coating, followed by drying at 250 °C for 5 min. Each sample was coated twice using the same procedure. The deposited HfO_2_ thin films on p-Si wafers were annealed at 600 °C for 30 min to obtain the m-HfO_2_ phase,^44^ which resulted in HfO_2_/p-Si structures. One of the samples was selected as a reference point and named H2.

Then, to create the ZnO layer, we prepared the ZnO sol. For this, ZnAc was dissolved in methanol and then NaOH was added. The ZnO sol was mixed on a magnetic stirrer at 60 °C for 1 h. The ZnO sol was deposited on the other three HfO_2_-coated samples at 500 rpm for 5 s and then at 3000 rpm for 30 s, then dried at 130 °C for 5 min. We repeated this procedure separately for the three remaining samples: one sample was coated once and named as H2Z1, another was coated 3 times and named H2Z3, and the final sample was coated 5 times and named H2Z5. Then, the deposited ZnO thin films were annealed at 500 °C for 60 min to obtain the w-ZnO phase,^45^ which resulted in three ZnO/HfO_2_/p-Si structures/The entire experimental process is summarized in Figure S1.

### 2.2 Thin-Film Characterization

XRD spectra of the obtained films were measured using the BRUKER D8 Advanced XRD instrument in the range of 2θ = 20–80° with 0.02° steps. The measurements were conducted with monochromatic Cu Kα radiation (λ = 1.5406 Å). The films were investigated using the HITACHI SU8700 for FE-SEM images and the Oxford Multimax100 detector for EDS spectra. The images were captured under high vacuum conditions without the need for Au coating. AFM images of thin films were investigated by Nano Magnetics. The images were captured on a 30 μm × 30 μm area. The optical properties of the films were investigated using the Shimadzu UV-1800 UV-visible (UV–vis) Spectrophotometer.

### 2.3 Device Fabrication and Characterization

**Figure 1:**
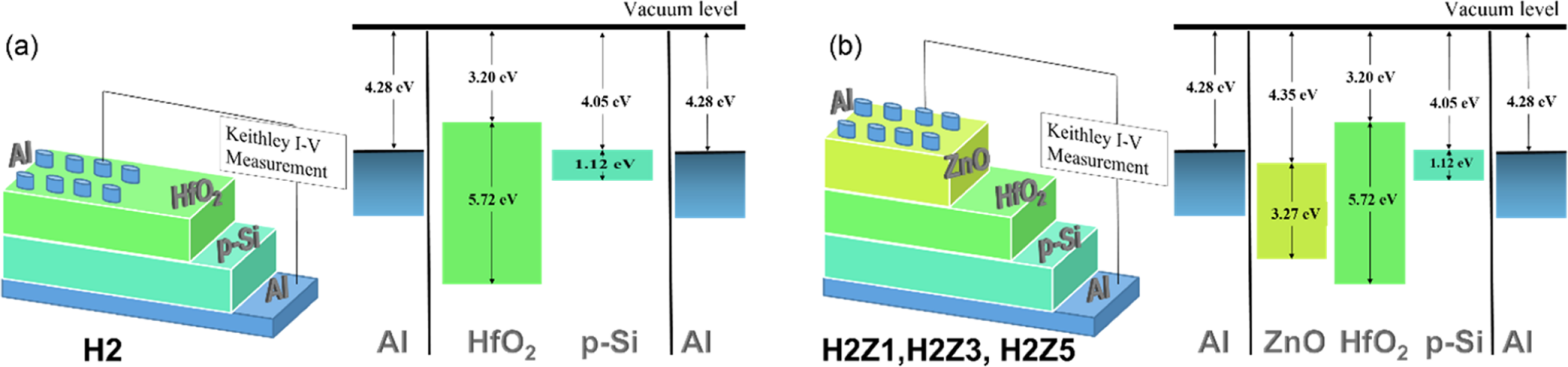
Schematic diagrams and energy band diagrams of devices with single-layer and multilayer structures: (a) H2 and (b) H2Z1, H2Z3, H2Z5.

For electrical measurements, the bottom faces of the samples were cleaned with HF and rinsed with deionized water. Aluminum (Al) was evaporated on all bottom faces of all four samples at a pressure of 10^–5^ Torr. The Al-covered samples were then rapidly sintered at 570 °C for 5 min in an Ar atmosphere to obtain ohmic contacts. After forming the bottom ohmic contacts, top contacts were created by evaporating Al dots with a diameter of 2.25 mm on the top faces of all four samples. Finally, Schottky-like structures were fabricated, specifically Al/HfO_2_/p-Si/Al (H2) and Al/ZnO/HfO_2_/p-Si/Al (H2Z1, H2Z3, H2Z5). Copper wires were connected to the aluminum on both sides on the films using silver paste. Electrical measurements (*I*–*V*) were conducted using two contact techniques. These measurements for all samples were carried out in the voltage range from −4 to 4 V step by 0.01 V at dark condition using a Keithley 2400 Source Meter. Figure 1 shows the schematic cross sections and energy band diagrams for the devices, as described in the Experimental Section. Figure 1a represents the sample H2, while Figure 1b displays the samples H2Z1, H2Z3, and H2Z5.

## 3 Results and Discussion

### 3.1 Characterization of Thin Films

**Figure 2:**
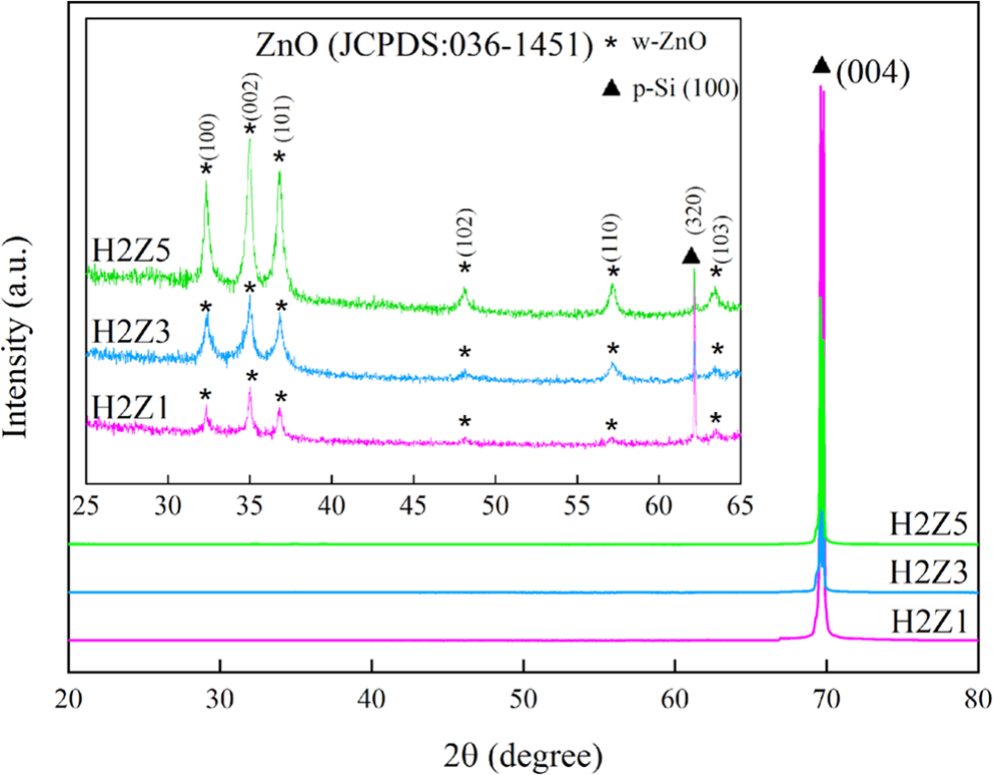
XRD pattern of multilayer ZnO/HfO_2_ structures.

**Figure 3:**
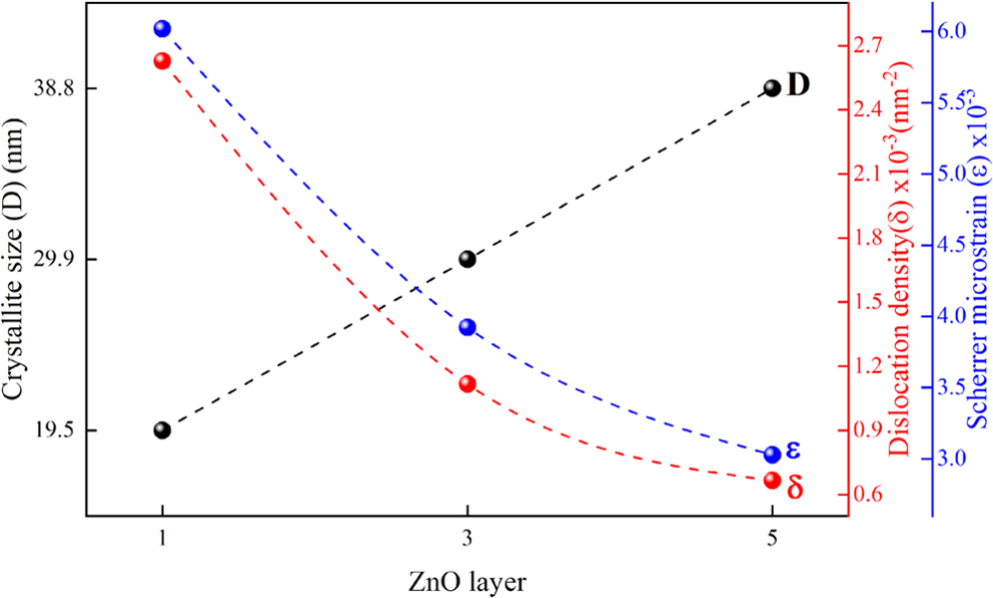
Variation of XRD parameters of ZnO/HfO_2_ multilayer structures with layer thickness.

XRD patterns and crystallographic properties of ZnO/HfO_2_/p-Si and HfO2/p-Si structures and the effect of increasing coating thickness were investigated. Initially, a dominant Si peak is observed at 2θ, 69°, attributed to the underlying substrate in Figure 2. As the coating thickness increases, the intensity of this Si peak gradually diminishes, indicating a reduction in the influence of silicon. On closer inspection the inset chart ranges from 25 to 65°. An increase in ZnO coating thickness corresponds to a notable enhancement in the intensity of ZnO peaks, progressively becoming more distinct. Conversely, no significant contribution from HfO_2_ is observed in the XRD patterns, which aligns with expectations, considering its approximate thickness of 20 nm.^46,47^ Using XRD spectrum data, structural parameters such as crystallite size (*D*), dislocation density (δ), and deformation in the crystal structure (microstrain (ε)) can be calculated via eqs 1–3. *D* can be determined using the Scherrer formula provided below.^48^
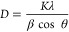
1In eq 1, *D* is the average crystallite size, *K* is the Scherrer constant (typically around 0.9), λ is the wavelength of the X-rays used, β is the full width at half-maximum (fwhm) of the XRD peak, θ is the Bragg diffraction angle. This equation provides an estimate of the average size of the crystalline domains in a material based on the broadening of the XRD peaks. Linear defects in the crystal structure, known as dislocations, are dependent on the morphological characteristics of the material and the formation and size of particles. They directly affect the material’s strength, hardness, and ductility. To quantify the amount of these defects, the expression for dislocation density provided below is employed.
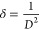
2
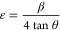
3Crystal size values of the samples, calculated using the Scherrer equation, exhibit a direct correlation with coating thickness. As seen in Figure 3 and Table 1, the changes in dislocation density and Scherrer microstrain are opposite to the changes in crystallite size. The increase in ZnO layer thickness leads to an increase in *D*, which is due to the coalescence of crystallites and, consequently, the improved crystallinity of the films.^49−51^ The dislocation density, indicative of the defect concentration in the film, is calculated using the eq 2.^52^ These values are presented in Table 1. It is observed that the increase in ZnO layer thickness leads to a decrease in dislocation density. The dislocation density varied from 2.62 × 10^–3^ to 1.66 × 10^–3^ with variations due to reduced defects between interfaces of the grains caused by the decreased oxygen vacancies.^22^Figure 3 shows that the Scherrer microstrain (ε) decreases as the ZnO film thickness increases. This reduction in Scherrer microstrain may be attributed to the predominant recrystallization process in the polycrystalline films and the migration of interstitial Zn atoms from within the crystallites to the grain boundaries, leading to a decrease in lattice imperfections. This occurs because the crystallinity of the films improves and the grain size increases as the film thickness grows.^53^ As the thickness of the ZnO thin films increased, the intensity of the (002) diffraction peak grew stronger while the fwhm decreased, as depicted in Figure 2. This suggests an improvement in the crystal quality of the films. A narrower fwhm of the (002) diffraction peak of ZnO films was closely related to both grain size and crystal quality.^54^ The use of high-quality ZnO thin films as active layers in applications may improve the quality and the lifetime of the applications.

**Table 1:** Variation of XRD Parameters of ZnO/HfO_2_ Multilayer Structures with Layer Thickness

sample	crystal size (*D*) (nm)	dislocation density (δ) (nm^–2^) × 10^–3^	Scherrer microstrain (ε) × 10^–3^
H2Z1	19.51	2.62	6.01
H2Z3	29.93	1.11	3.92
H2Z5	38.77	0.66	3.03

**Figure 4:**
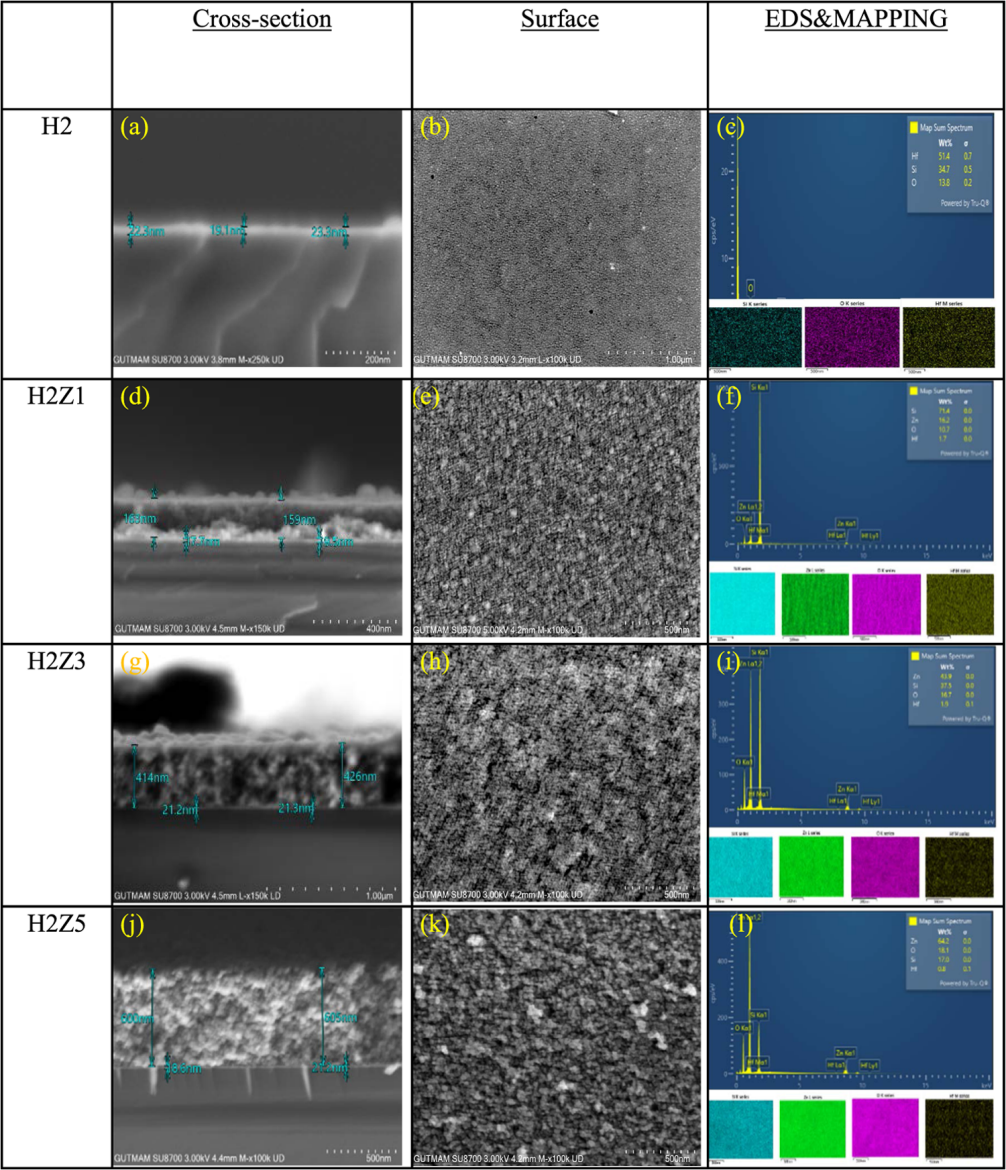
Cross-sectional FE-SEM images, surface morphology, and EDS mapping results for HfO_2_ and multilayer ZnO/HfO_2_ structures: (a, d, g, j): Cross-sectional FE-SEM images of the H2, H2Z1, H2Z3, and H2Z5 samples, respectively, showing the structural composition and layer thicknesses. (b, e, h, k): Surface FE-SEM images illustrating the surface morphology of the corresponding samples. (c, f, i, l): EDS mapping results showing the elemental distribution across the surface of the H2, H2Z1, H2Z3, and H2Z5 samples, respectively.

All the cross-sectional and surface FE-SEM images, along with the EDS and mapping results for the samples, are presented in Figure 4 and Table 2. As shown in Figure 4, the cross-sectional images indicate that the thickness of the HfO_2_ layer is approximately 20 nm in all samples. The thicknesses of the 1, 3, and 5-layer ZnO coatings are 160 nm in Figure 4d, 420 nm in Figure 4g, and 602 nm in Figure 4j, respectively. The total thicknesses of the samples H2, H2Z1, H2Z3, and H2Z5 are determined to be 20, 180, 440, and 622 nm, respectively. In Figure 4b, the H2 surface exhibits a homogeneous and well-organized structure. The presence of smaller grains indicates an increased number of grain boundaries. Conversely, in Figure 4k, the effect of increased ZnO layer thickness results in agglomerations and larger grains, which correspond to a reduction in the number of grain boundaries. Thus, it can be stated that the effect of the grain boundary decreases with the increasing thickness of the ZnO layer. Additionally, as can be seen from the EDS results in Figure 4, as the ZnO coating thickness increases, the elemental percentage of Zn increases, while the elemental percentage of Hf remains nearly constant.

**Table 2:** Results Obtained from UV, AFM, FE-SEM, and EDS Spectrum for all Samples

	UV	AFM		FE-SEM		EDS			
	*E*_g_ (eV)	RMS	peak to peak	HfO_2_ thickness (nm)	ZnO thickness (nm)	O (wt %)	Si (wt %)	Hf (wt %)	Zn (wt %)
H2	5.59	0.52	6.38	20 ± 3		3.5	90.4	6.2	
H2Z1	4.15	5.17	46.67	20 ± 3	160 ± 3	12.9	52.8	4.6	29.5
H2Z3	4.29	3.74	31.00	20 ± 3	420 ± 3	16.7	37.5	1.9	43.9
H2Z5	4.38	1.87	17.70	20 ± 3	602 ± 3	18.1	17	0.8	64.2

**Figure 5:**
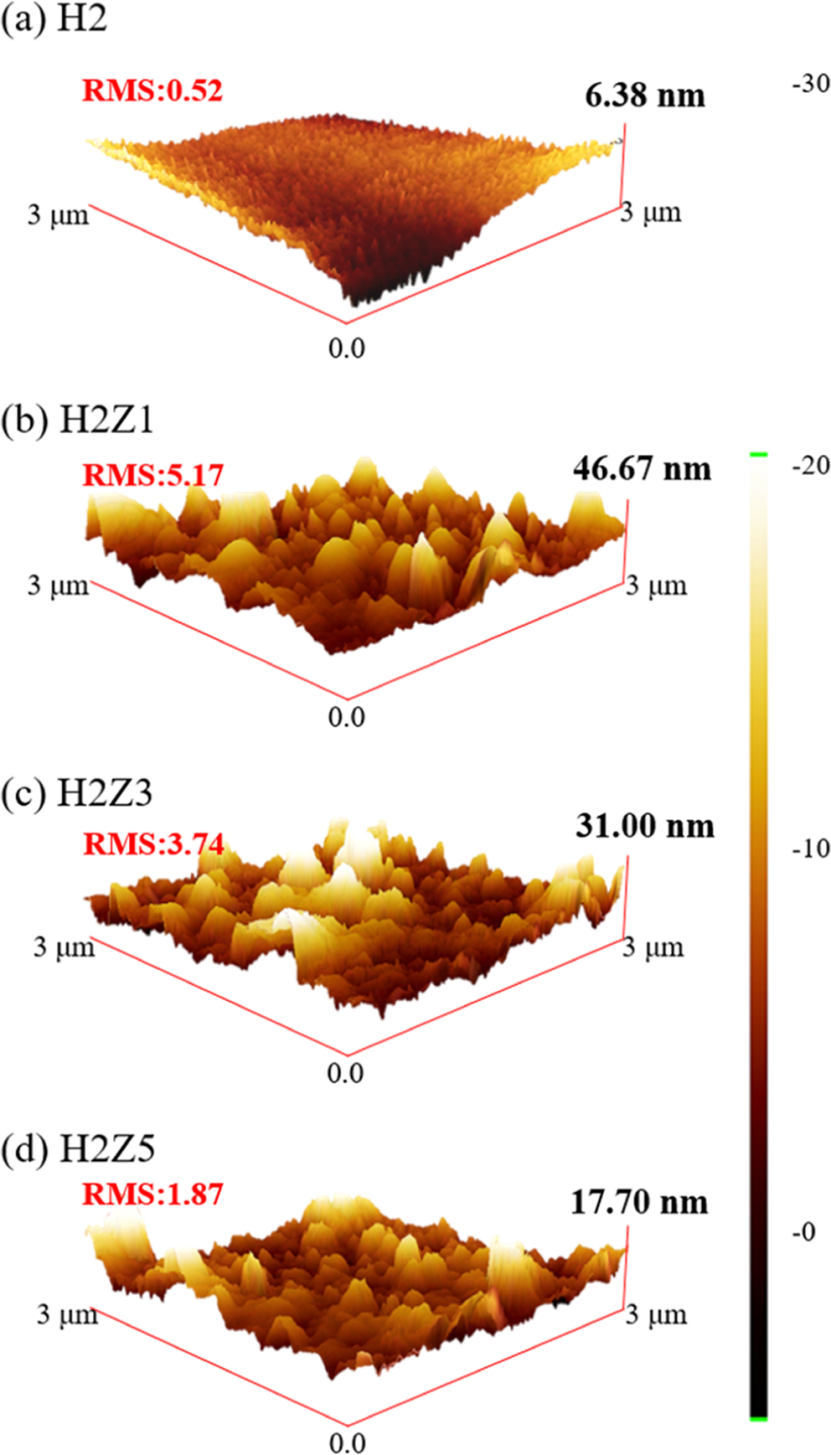
AFM images and surface roughness of HfO_2_ and multilayer ZnO/HfO_2_ structures: (a) H2, (b) H2Z1, (c) H2Z3, (d) H2Z5.

From the AFM images in Figure 5, it can be observed that voids and particles are homogeneously distributed on the surface. The HfO_2_ layer appears to be quite homogeneous with small grains. However, with the addition of one layer of ZnO, the structures become significantly larger. As the ZnO layer increases from one to three layers, the average surface roughness (RMS) decreases. Similarly, the peak-to-peak values of the films initially increased noticeably with the first ZnO layer, then decreased slowly as more ZnO layers were added. For samples H2, H2Z1, H2Z3, and H2Z5, RMS and the peak-to-peak values are provided in the Table 2.

**Figure 6:**
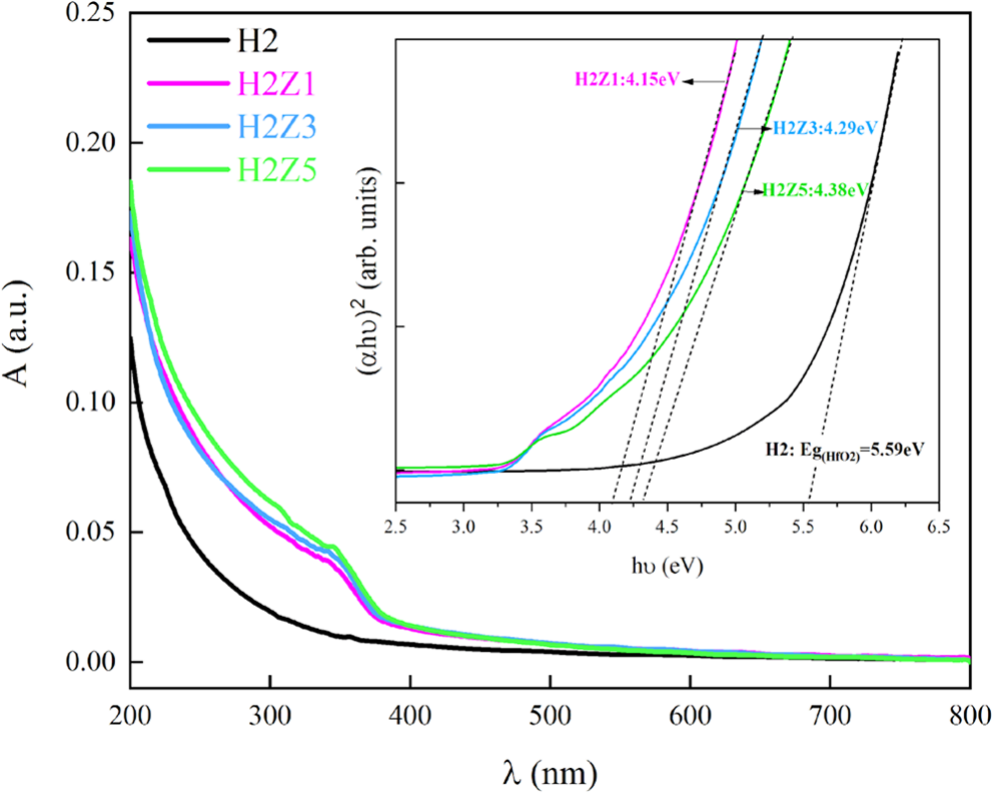
Optical band gaps from UV spectrum of HfO_2_ and multilayer ZnO/HfO_2_ structures.

The band gap value of HfO_2_ and multilayer ZnO/HfO_2_ structures was determined by Tauc’s plot.
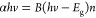
4Here, *E*_g_ and *h*υ are the optical band gap and incident photon energy, respectively. While *B* is an energy-independent constant, *n* is an indicator for the nature of the band gap (*n* = 1/2, allowed direct band gap; *n* = 3/2, allowed indirect band gap). In determining the optical band gaps of ZnO and HfO_2_, an *n* value of 1/2 was used. In Figure 6 of the absorption spectrum, the samples coated with ZnO show enhanced light absorption intensity in the range of 350–500 nm compared to the sample coated only with HfO_2_ (H2).

The inset of Figure 6 and Table 2 shows the optical band gap of multilayer films calculated using the Tauc plot method (eq 4). The band gap values obtained for ZnO and HfO_2_, 3.32 and 5.59 eV respectively, are consistent with the literature.^47,55^ A shift in the band gap was observed with the increase in coating thickness of the multilayer films. This shift can be explained by the Burstein–Moss effect.^56,57^ The thickness of the ZnO layer increases, while the number of free charge carriers also increases. The increased free charge carriers fill the states near the bottom of the conduction band, leading to a shift in the Fermi level and resulting in the widening of the band gap.

### 3.2 Electrical Properties of HfO_2_ and Multilayer ZnO/HfO_2_ Devices

**Figure 7:**
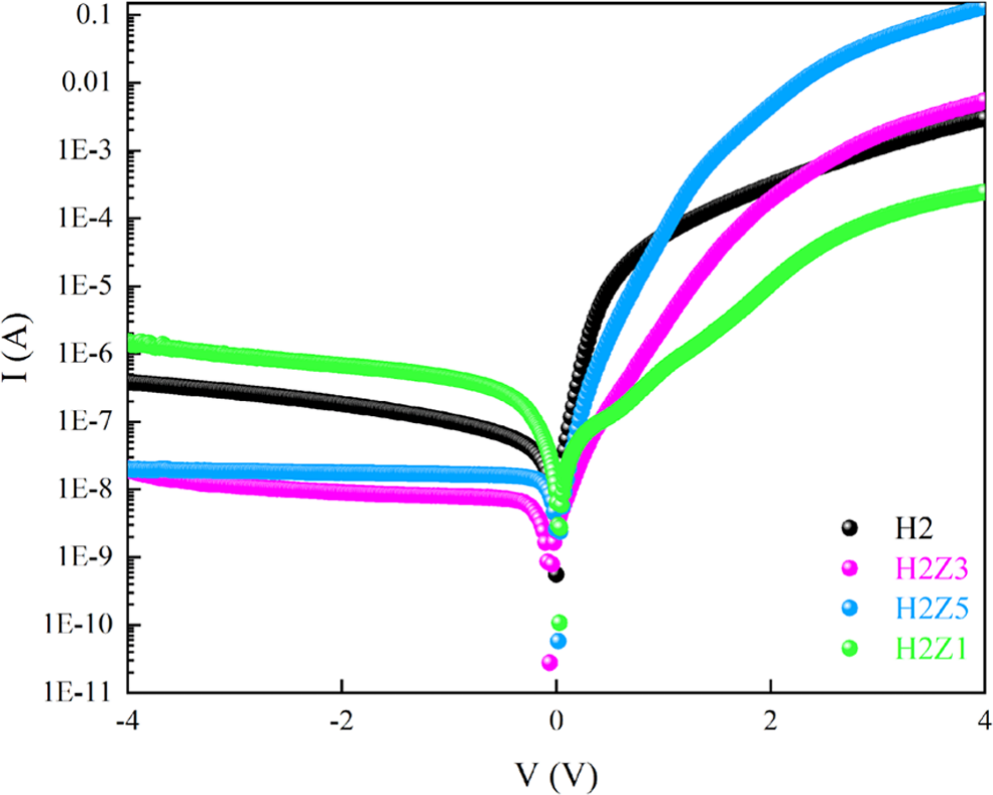
ln *I*–*V* plots of HfO_2_ and multilayer ZnO/HfO_2_ devices.

Measurements of current voltage offer essential information into heterojunction systems containing interface layer. The ln *I*–*V* plot characteristics for each sample are illustrated in Figure 7. It is clear from the figures that the curves of all samples show diode-like behavior. In other words, the produced heterojunction diodes have asymmetric *I*–*V* characteristics, which is evidence of the formation of a rectifying junction of the Schottky type. For every sample, the rectification ratio (RR), or the ratio of the forward current to the reverse current, was computed at ∓4 V. RR values of *n*-ZnO/p-Si heterojunction diodes with HfO_2_ interface layer were calculated and revealed an excellent rectification characteristic of heterojunctions with rectification ratio of 2.65 × 10^5^ and 6.56 × 10^6^ for H2Z3 and H2Z5 samples, respectively, except for H2Z1 sample (RR = 163). This value was also calculated as 7.79 × 10^3^ for HfO_2_/p-Si (H2 sample) diode. As can be seen, the heterojunction diodes potential barrier formation may be the cause of the high RR values. The *n*-ZnO/p-Si heterojunction diodes with HfO_2_ interlayer appear to have a beneficial interface and strong device performance based on the comparatively high values of the rectification ratio.

The following formula can be used to express current (*I*) for heterojunctions and MS/MIS/MOS type diodes based on applied voltage:^58,59^
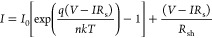
5where *I*_0_, *n*, *V* and *R*_sh_ are the saturation current, the ideality factor of diode, the voltage drops on the diode and the shunt resistance, respectively. The *IR*_s_ parameter in the second part of eq 5 shows the voltage falling on the series resistance.

As illustrated in Figure 7, the reverse bias current is practically voltage-independent for all samples, but in the forward bias area, the forward bias current value rises nearly exponentially with applied bias voltage. Due to the presence of the interface layer and series resistance at each sample, the ln *I*–V plot deviates from linearity at sufficiently high forward bias voltages. The first voltage region (0.08–0.3 V) for all samples and the second voltage region (0.4–2.2 V) for H2Z1, (0.4–1.5 V) for H2Z3, and (0.4–1.2 V) for H2Z5 samples are clearly represented by the two separate linear regions with varied slopes seen in Figure 7. The two parallel diode model can be identified for their behavior in the ln *I*–*V* plot for H2Z1, H2Z3 and H2Z5 samples. In the present case, the forward bias region for the relation between current and voltage in eq 1 can be rewritten as follows:^60−62^
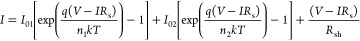
6The saturation currents (*I*_01_*and I*_02_), the ideality factors (*n*_1_*and n*_2_) and barrier heights (Φ_b1_ and Φ_b2_) were calculated by the following equations:^63−65^
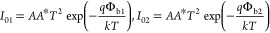
7
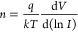
8
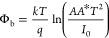
9where A* is the effective Richardson constant (that takes the value of 32 A/cm^2^ K^2^ for p-Si^43^ and *A* is the effective area of diodes.

According to thermionic emission (for *V* ≥ 3 *kT*/*q*) at first and second voltage regions, the slope and intercept of the ln *I*–*V* plots of Figure 7 are used to obtain the identity factor (*n*) and barrier height (Φ_b_), respectively. The calculated *n*, *I*_0_ and Φ_b_ values at first and second voltage regions for all samples were introduced in Table 3. In comparison to the HfO_2_/p-Si diode, Table 3 shows that the barrier height is higher in samples *n*-ZnO/p-Si with HfO_2_ interface layer. This growing tendency is maintained as the ZnO thickness increases. The H2Z5 sample has the largest barrier height value, 0.817 eV. The Sah-Noyce-Shockley theory^62^ for heterojunctions and thermionic emission theory for diodes define that *n* depends on the bias voltage. Typically, *n* is more than 2 (calculated value) for high voltage and equal to 1 for the ideal case.^65^ The two likely reasons for the deviation of *n* from unity are either recombination of electrons and holes in the depletion area or a rise in the diffusion current as a result of increasing the applied voltage. In addition to, for the produced samples, an ideality factor larger than 2.0 denotes a nonideal diode.^66,67^ The existence of defect states in the ZnO/HfO_2_ thin films or nonlinear metal–semiconductor contact^68−70^ may be the cause of the high ideality factor in this present work.

**Table 3:** Calculated Fundamental Experimental Parameters of the HfO_2_ and Multilayer ZnO/HfO_2_ Devices for First and Second Voltage Regions

	region I			region II		
sample	*I*_01_ (A)	*n*_1_	φ_b_ (eV)	*I*_02_ (A)	*n*_2_	φ_b_ (eV)
H2	9.36 × 10^–9^	2.155	0.780			
H2Z1	4.94 × 10^–9^	2.838	0.796	2.90 × 10^–8^	13.205	0.750
H2Z3	2.86 × 10^–9^	4.003	0.810	1.17 × 10^–8^	7.222	0.774
H2Z5	2.24 × 10^–9^	2.124	0.817	5.92 × 10^–8^	5.603	0.732

**Figure 8:**
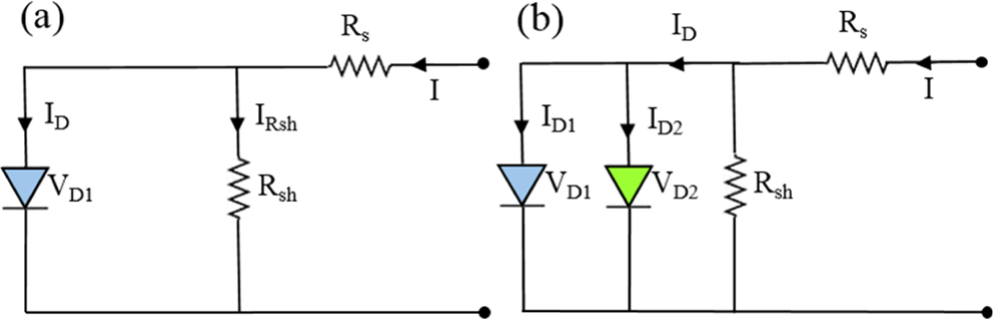
Schematic diagram of circuit model for (a) H2 sample (b) H2Z1, H2Z3 and H2Z5 samples.

Figure 8 shows the analogous circuits for one diode model and two parallel diodes. Furthermore, the diffusion and recombination generation current components, respectively, may be defined by the first and second terms in eq 6.^68^ As can be seen from Figure 8, H2 sample fit one diode model and H2Z1, H2Z3 and H2Z5 samples fit two parallel diode models.

Figure 7 shows the experimental reverse *I*–*V* curves plotted for all the samples. As can be seen Figure 7, the reverse leakage current (*I*_rlc_) of the *n*-ZnO/p-Si heterojunction diodes with HfO_2_ thin film decreases as a function of reverse bias increase. Meanwhile, a low-reverse leakage current is a critical requirement for the use of heterojunction diodes in electronic device applications. Furthermore, when calculating the power loss for diode applications, the *I*_rlc_ is also crucial. The values of *I*_rlc_ for the H2, H2Z1, H2Z3 and H2Z5 samples were calculated as 3.82 × 10^–7^, 1.49 × 10^–6^, 2.04 × 10^–8^ A and 2.02 × 10^–8^*I*_rlc_ value is significantly influenced by the HfO_2_ insulating layer. In other word, the interfacial defects can be decreased when the HfO_2_ interface layer is deposited between the *n*-ZnO/p-Si heterojunction diodes. This will significantly improve the interfacial structure and lower the leakage current. One possible explanation for the larger *I*_rlc_ for H2 and H2Z1 is that there are more minority charge carriers traveling through the tunnel.^69^ Interestingly, the H2Z3 and H2Z5 samples reveal the lowest *I*_rlc_ values of all the samples. This phenomenon is due to the homogeneous distribution of the electric field and the less of defects in the H2Z3 and H2Z5 samples. Furthermore, it is noteworthy that the reverse current also decreases as ZnO thickness increases. These findings show that reducing *I*_rlc_ through the deposition of more *n*-ZnO thickness in *n*-ZnO/p-Si heterojunction diodes with a HfO_2_ interface layer can enhance diode performance. Here, since the current is insufficient to provide enough electrons into the space-charge region, tunneling current in the low-reverse bias range determines the *I*–*V* characteristics in our cases. In contrast, SCLC begins to predominate under the high-reverse level when the tunneling current grows to a level where it can supply enough electrons into the depletion region.

**Figure 9:**
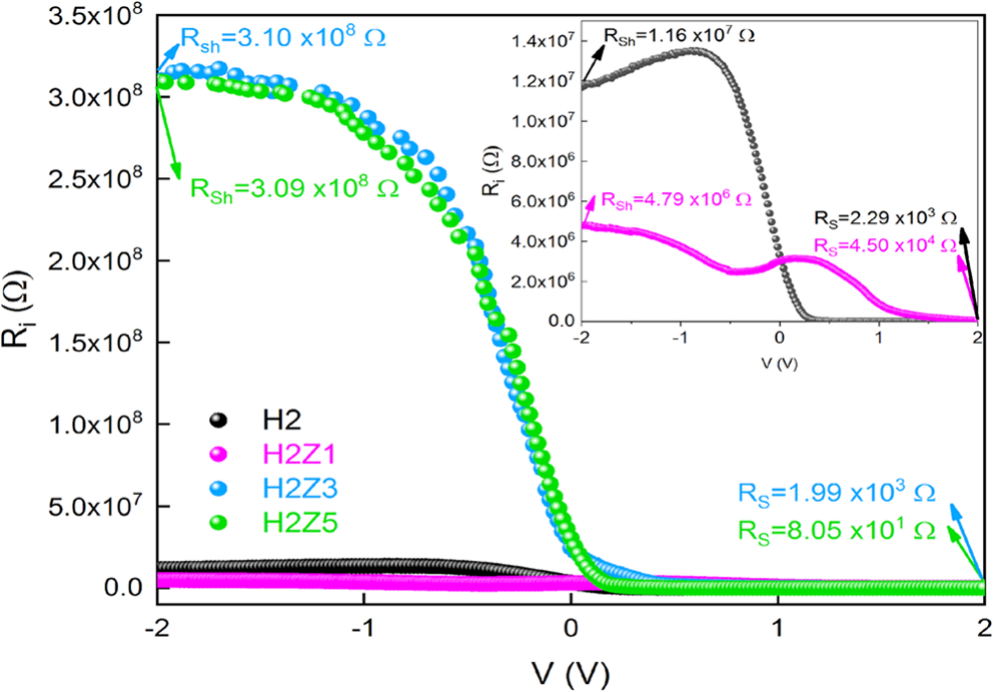
*R*_*i*_-*V* plots of the HfO_2_ and multilayer ZnO/HfO_2_ devices.

The crucial factors affecting the efficiency of heterojunction diodes and Schottky diodes are the magnitude values of the series resistance (*R*_s_) and shunt resistance (*R*_sh_) shown in the circuit diagram of Figure 8. Using the  equation, we determined the values of *R*_s_ and *R*_sh_ for each sample and showed them in the *R*_*i*_-*V* plots in Figure 9. Figure 9 shows that the values of *R*_sh_ is obtained from the resistance value *R*_*i*_ at the lowest negative voltage and the values of *R*_s_ is obtained from the resistance value *R*_*i*_ at the highest positive voltage. According to Figure 9, for example, the values of *R*_s_ and *R*_sh_ were determined as 2.29 × 10^3^ Ω and 1.16 × 10^7^ Ω for H2 sample and 80.5 Ω and 3.09 × 10^8^ Ω for H2Z5 and at ∓2 V. It has been observed that *R*_sh_ increases and *R*_s_ decreases as the ZnO thickness increases for the fabricated samples.

**Figure 10:**
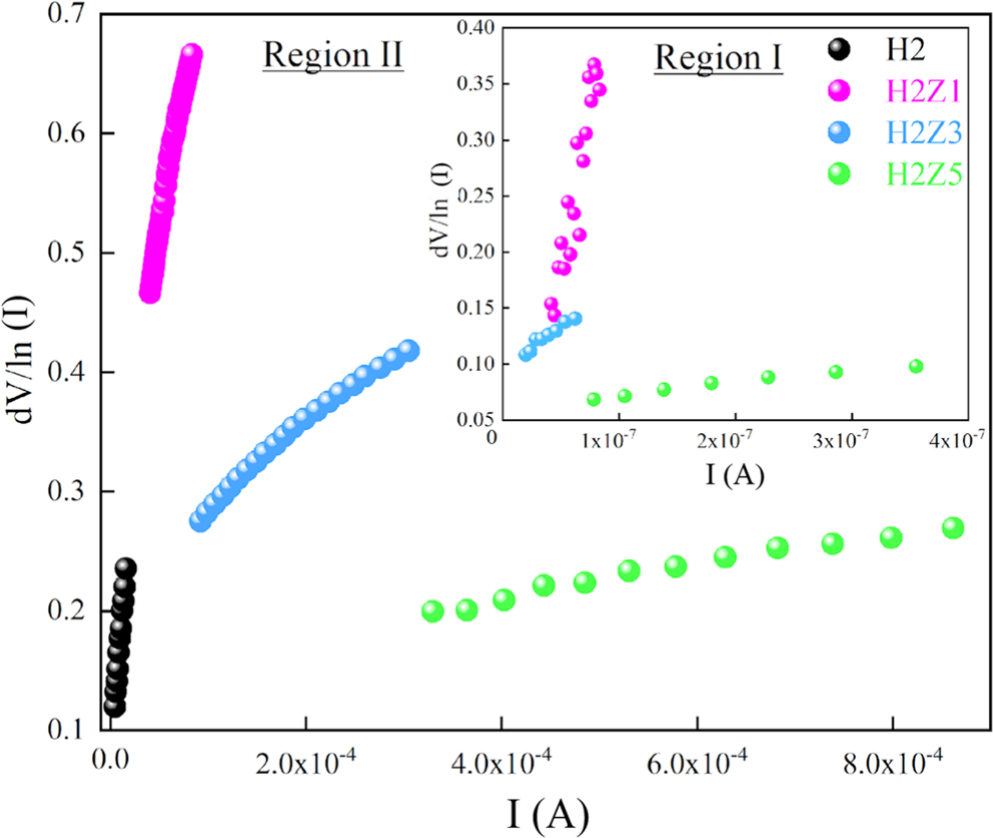
d*V*/d(*ln I*)—*I* plots of the HfO_2_ and multilayer ZnO/HfO_2_ devices.

**Figure 11:**
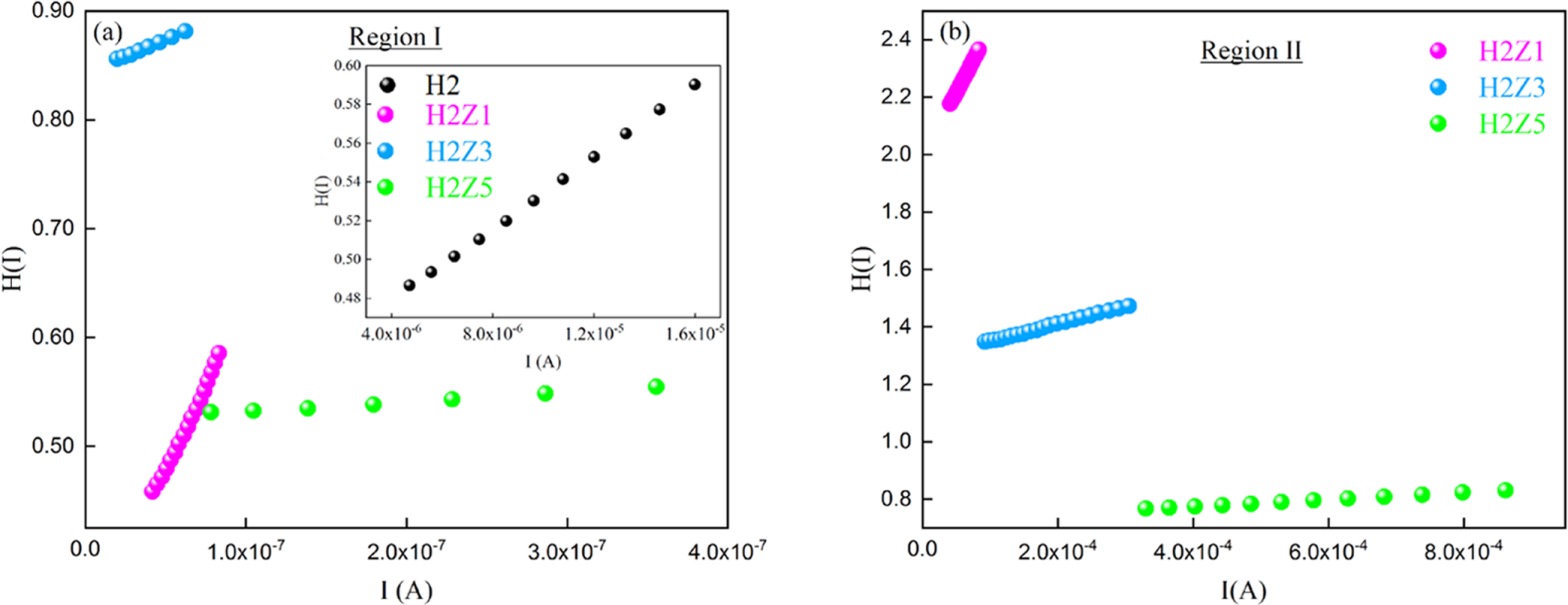
*H*(*I*) vs *I* plots of the HfO_2_ and multilayer ZnO/HfO_2_ devices; (a) region I (b) region II.

Another alternative way to determine *R*_s_ values is to use the Cheung–Cheung method.^70^ This method allows for the calculation of *n* and Φ_b_ values in addition to *R*_s_ computation in the series resistance region at high voltage values. The following equations can be used to characterize the quantities of the  and *H*(*I*) functions of the Cheung and Cheung method, respectively.^70−73^
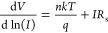
10
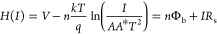
11For the HfO_2_/p-Si diode and *n*-ZnO/p-Si heterojunction diodes with HfO_2_ interlayer, the experimental values of series resistance (*R*_s_) were determined using eq 10 and the d*V*/d(*ln I*)—*I* plot illustrated in Figure 10. The *R*_s_ is represented by the slope of the straight-line fit on the plot of d*V*/d(*ln I*) vs *I*, and the value of *n* is found at the intercept. Their values are given in Table 3. The values of *n* determined from the d*V*/d(*ln I*)—*I* plot change between 1.848 and 3.856 in *n*-ZnO/p-Si heterojunction diodes with HfO_2_ interface layer. In general, the values of ideality factor (*n*) have far from ideal behavior due to the presence of interface states and series resistance, a significant probability of hole and electron recombination in the depletion zone and barrier inhomogeneities.^74,75^ Meanwhile, in the first diode region, series resistance values in heterojunction diodes with HfO_2_ were obtained much higher than in HfO_2_/p-Si (H2) diode. Series resistance values approximately in the MΩ range were obtained in *n*-ZnO/p-Si heterojunction diodes with HfO_2_. For all samples, the *R*_s_ values were also found using eq 11 and the *H*(*I*)—*I* plot Figure 11. The *R*_s_ is found by the slope of the straight-line fit on the plot of *H*(*I*) vs *I*, and the value of Φ_*b*_ is determined at the intercept. Their values are given in Table 4. The values of Φ_*b*_ determined from the *H*(*I*)—*I* plot change between 0.145 and 0.219 eV in *n*-ZnO/p-Si heterojunction diodes with HfO_2_ interface layer and HfO_2_/p-Si diode.

**Table 4:** Calculated Fundamental Experimental Parameters of the *n*-ZnO/p-Si Heterojunction Diodes with HfO_2_ Interface Layer and HfO_2_/p-Si Diode for First and Second Voltage Regions

	region I				region II			
	d*V*/d*lnI*–*I*		*H*(*I*)–*I*		d*V*/d*lnI*–*I*		*H*(*I*)–*I*	
sample	*R*_s1_ (Ω)	*n*_1_	φ_b1_ (eV)	*R*_s1_ (Ω)	*R*_s2_ (Ω)	*n*2	φ_b2_ (eV)	*R*_s2_ (Ω)
H2	10.05 × 10^3^	3.056	0.145	9.23 × 10^3^				
H2Z1	4.74 × 10^6^	1.848	0.174	3.12 × 10^6^	4690	11.44	0.174	4468
H2Z3	7.41 × 10^5^	3.856	0.219	6.07 × 10^5^	670	8.896	0.145	607
H2Z5	1.08 × 10^5^	2.464	0.213	8.67 × 10^4^	135	6.292	0.155	121

One of the variables that will affect the stability and performance of heterojunction diodes is *R*_s_. Another method to determine *R*_s_ in these devices is the modified Norde function for *n* > 2. This function indicates that the values of *R*_s_ and Φ_b_ can be found by locating the minimum point on the *F*(*V*)—*V* curve near the concave curve of the *ln I*–*V* curve. The following improved Norde equations can be used to find *R*_s_ and Φ_b_ values:^76−78^
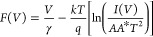
12
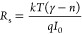
13
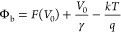
14where the first integer bigger than *n* is γ. *F*(*V*_0_) is the *F*(*V*)’s least value. *V*_0_ and *I*_0_ are the values of voltage and current corresponding to this least value, respectively.

As shown in Figure 11, the intercept and slope of this model were used to fit the *H*(*I*) vs *I* graph linearly, yielding Φ_b_ and *R*_s_, whose values are shown in Table 4. As can be shown in Table 5, it was remarkable to note that the barrier height values increased with both HfO_2_ deposition and increasing n*-ZnO* thickness. There was good agreement between the barrier height parameters obtained from the Norde method and the *ln I*–*V* (Figure S2) method for all samples. As can be seen Tables 3 and 4, for all samples, the barrier height parameters obtained from the ln *I*–*V* method are in good agreement with the modified Norde method. This is because the diode parameters are calculated for both methods in the thermionic emission region at low voltages in this study. Meanwhile, the utilization of distinct regions of *I*–*V* characteristics accounts for the variation in barrier height between the Norde and Cheung methods.

**Table 5:** Experimental *R*_s_ and Φ_b_ Values Obtained from the Norde Method for all Samples

	*F*(V)–*V*	
sample	φ_b_ (eV)	*R*_s_ (Ω)
H2	0.779	1.44 × 10^4^
H2Z1	0.794	1.85 × 10^5^
H2Z3	0.809	2.71 × 10^5^
H2Z5	0.814	1.24 × 10^5^

The barrier height and ideality factor values obtained in this study demonstrate superior performance compared to similar MIS and MOS structures reported in the literature. For instance, the barrier height obtained in the Al/ZnO/HfO_2_/p-Si heterostructure shows a significant increase, reaching 0.81, compared to previously reported values of 0.77^79^ 0.66,^80^ 0.79,^81^ and 0.69^82^ for some MIS structures. This difference is attributed to the optimized deposition process and high crystal quality of the ZnO and HfO_2_ layers.

Moreover, the ideality factor obtained in this study also shows a significant decrease, reaching 2.12, compared to previously reported values of 2.81,^83^ 4.74^84^ 3.72^85^ and 6.37^86^ for some MOS structures. This lower ideality factor indicates less recombination at the interface and better electrical properties. These results highlight not only the high quality of the materials and fabrication methods used but also demonstrate that this study presents a heterostructure with better performance compared to existing studies in the literature.

**Figure 12:**
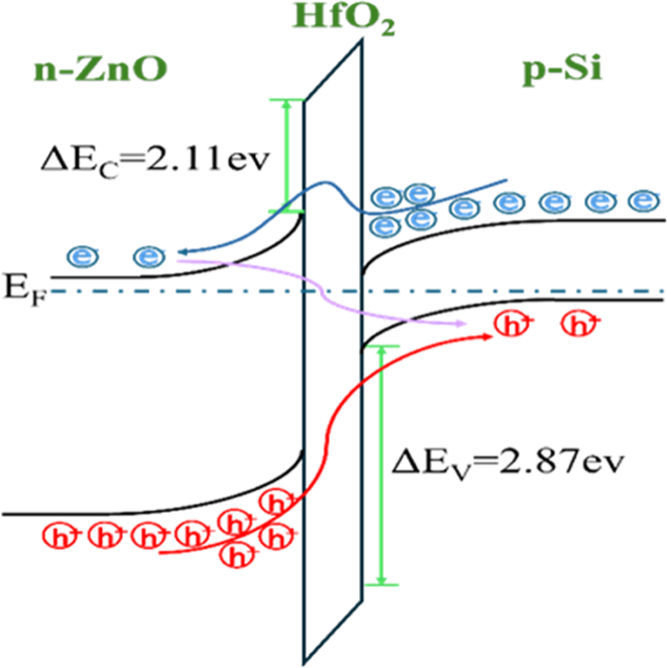
Energy band structure diagram for *n*-ZnO/HfO_2_/p-Si heterojunction with forward applied voltage and the carriers transport.

Figure 12 illustrates the carrier motions occurring under forward bias in the *n*-ZnO/p-Si heterojunction and the influence of the HfO_2_ barrier on these movements. Electrons move from the conduction band of *n*-ZnO to the conduction band of p-Si (path blue), while holes attempt to transfer from p-Si to *n*-ZnO (path arrow). However, the HfO_2_ barrier imposes energy barriers for both carriers, limiting their flow. Additionally, electrons in *n*-ZnO recombine with holes in p-Si through the interface states (path purple), negatively affecting the forward current. Evaluating the effect of changes in ZnO thickness on the interface state density of the HfO_2_ barrier is crucial for optimizing the performance of the heterojunction.

**Figure 13:**
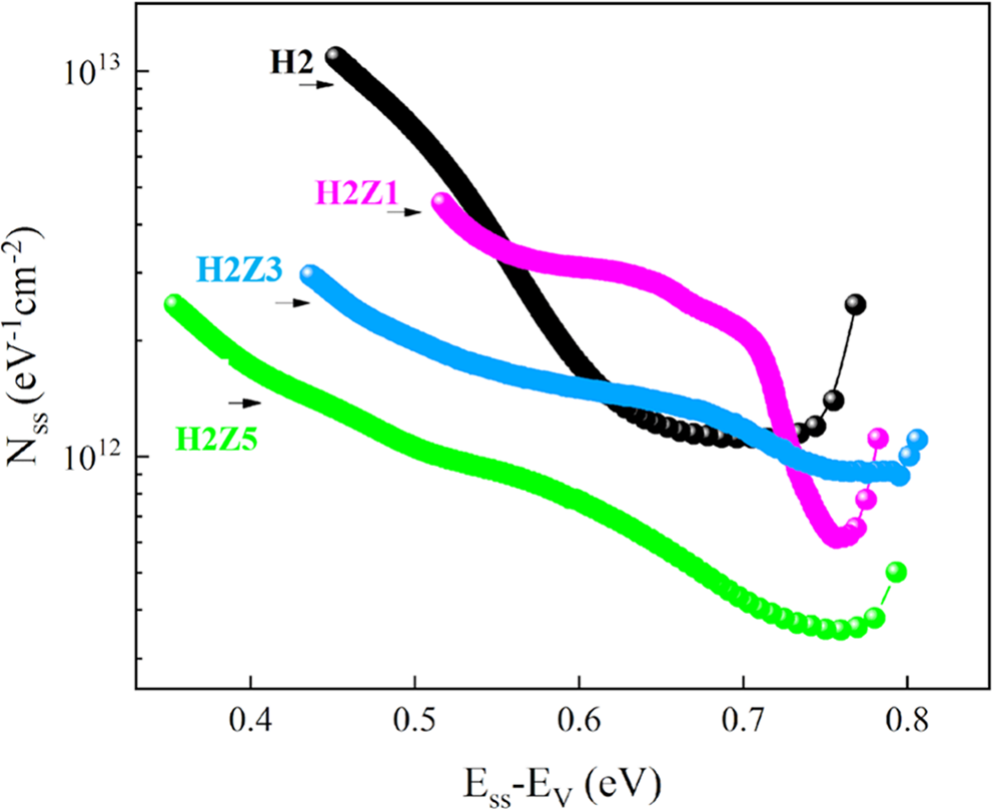
*N*_ss_ – (*E*_ss_ – *E*_V_) curves of the HfO_2_ and multilayer ZnO/HfO_2_ devices.

The distribution of interface states (*N*_ss_) between semiconductor and metal,^50,87^ or the interface layer,^76,77^ affects the speed, stability, and reliability of heterojunction diodes during operation. Therefore, knowing the distribution of interfacial states in the energy band gap of the semiconductor is especially important for its usability in devices.^88,89^ Card and Rhoderick^4^ proposed the *N*_ss_, interface states density, which is given following equation in its most basic form:^4,90^
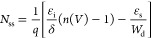
15Here, the interlayer’s thickness is represented by δ, while the semiconductor and interlayer permittivities are represented by ε_s_ and ε_i_, respectively. The energy distributions (*E*_ss_ – *E*_V_) in the *p* type semiconductor structures are expressed as,^91^
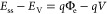
16The applied voltage, *V*, is given by  in this case. eqs 15 and 16 were utilized to compute interface state densities based on energy distribution, and Figure 13 displays the *N*_ss_ – (*E*_ss_ – *E*_V_) curves for all samples. In Figure 13, the *N*_ss_ for the *n*-ZnO/p-Si heterojunction diodes with HfO_2_ interface layer varied between 4.56 × 10^12^ and 5.01 × 10^12^ eV^–1^ cm^–2^. The experimental findings indicated that the *N*_ss_ for the H2Z5 sample increased exponentially range from 5.01 × 10^11^ eV^–1^ cm^–2^ in (0.793-*E*_V_) eV to 2.47 × 10^12^ eV^–1^ cm^–2^ in (0.354-*E*_V_) eV, whereas the estimated values of *N*_ss_ for the H2 sample increased exponentially range from 2.47 × 10^12^ eV^–1^ cm^–2^ in (0.768-*E*_V_) eV to 1.09 × 10^13^ eV^–1^ cm^–2^ in (0.452-*E*_V_) eV.

**Figure 14:**
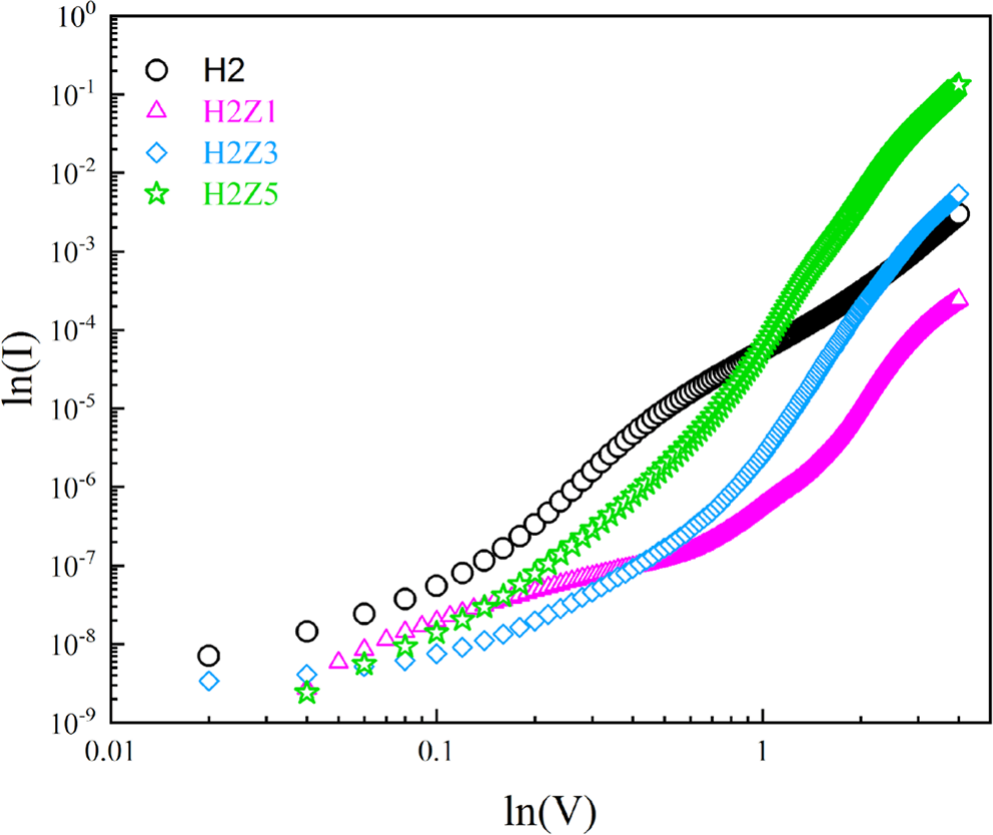
Plot of log(*I*) versus log(*V*) for the H2, H2Z1, H2Z3 and H2Z5 diodes.

As seen in Figure 14, the *I* vs *V* curves for the H2, H2Z1, H2Z3, and H2Z5 diodes are plotted in logarithmic scale under the forward bias to obtain a deeper knowledge of the charge transit processes. Four distinct regions are presented in the plot for the H2, H2Z1, and H2Z3 diodes with varying slopes. The slope at unity (*m*–1), where conduction is governed by the Ohmic behavior at low bias, is referred to as Region-I. A Schottky diode’s current is supposed to have a power law relationship with the applied voltage, such as *I*–*V^m^*, under the SCLC theory. According to this process, since the slope (*m*) is near to 2, the SCLC mechanism in Region III for the H2 diode, in Region II for the H2Z3 diode and in Region I for the H2Z5 diode is dominated by a shallow trap. In other diode regions, the SCLC is regulated by exponentially distributed traps.

The SCLC theory gives essential information about transportation mechanisms and mobility through interfaces of *n*-ZnO//HfO_2_/p-Si/Al heterojunction diode.^92,93^ At dominating SCLC region, effective mobility is calculated with helping Mott–Gurney SCLC equation,
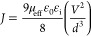
17***J*** is current density, ε_i_ is the dielectric constants of all diodes, ε_0_ is the permittivity of free space, and *d* is the thickness of H2, H2Z1, H2Z3, and H2Z5 heterojunction diode. Using the slope of *J*–*V*^2^ curve (Figure S3) and eq 17, μ_eff_ can be determined. Values of effective mobility for the diodes are given in Table 6. According to Table 6, effective mobility increased with adding *n*-ZnO layer, also these increased with increasing thickness of *n*-ZnO layer. Following this result, it can be said that the *n*-ZnO layer contributed to the increase in mobility, also the increase in the thickness of the *n*-ZnO layer may have increased in mobility. The diffusion length can also be calculated thanks to the Einstein–Smoluchowski equation.^94^
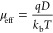
18*q* is charge of carrier, *D* diffusion coefficient and this value is unitless which depends on the morphology of the material,^81^*k*_b_ is Boltzmann constant, and *T* is temperature. The diffusion length is given in eq 19. Values of all parameters such as *D* and *L*_d_ are given in Table 6.
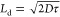
19Also, the transition time of charge carriers from the region dominated by the SCLC can be determined.^79^ The slope of the plot of current density versus voltage (Figure S4) and the transit time can be calculated using eq 20;^95,96^
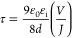
20The values of the transition time of *n*-ZnO//HfO_2_/p-Si/Al heterojunction diodes are given in Table 6. Dissimilar behavior in mobility is observed in the transition time. While the transit time decreased with the addition of the *n*-ZnO layer, these values increased with increasing layer thickness.

**Table 6:** Calculated Fundamental Experimental Parameters for SCLC Regions of the *n*-ZnO/p-Si Heterojunction Diodes with HfO_2_ Interface Layer and HfO_2_/p-Si Diode

	*J*–*V*^2^	*J*–*V*		
sample	μ_eff_	τ (s)	*D*	*L*_d_
H2	2.63 × 10^–7^	6.34 × 10^–5^	6.79 × 10^–9^	9.28 × 10^–7^
H2Z1	1.06 × 10^–6^	3.56 × 10^–8^	2.75 × 10^–8^	4.42 × 10^–8^
H2Z3	2.00 × 10^–4^	4.96 × 10^–7^	5.16 × 10^–6^	2.26 × 10^–6^
H2Z5	9.60 × 10^–3^	7.81 × 10^–6^	2.48 × 10^–4^	6.23 × 10^–5^

## 4 Conclusions

In this study, we used the sol–gel spin-coating method to produce the ZnO, HfO_2_, and ZnO/HfO_2_ interlayers between the metal and the p-Si crystal. By using XRD, AFM, FE-SEM, and EDX examine the structural and surface morphological characteristics of the interface layer (ZnO, HfO_2_, and ZnO/HfO_2_), we determined.

The electrical results exhibited an improvement in the diode parameter quality of the *n*-ZnO/p-Si heterojunction diodes with HfO_2_ interface layer. This enhancement in saturation currents (*I*_0_) and barrier heights (Φ_b_) values may also be attributed to the increased grain boundary effects, as observed from the SEM and AFM results.

Our results showed that the N_SS_ values of MIS SBDs are significantly lower than that of MS SBDs, suggesting that the number of dangling bonds decreases at silicon surface after inserting the Al_2_O_3_ interlayer.

We found that the power law *I*–*V^n^* relation may be used to estimate the *I*–*V* characteristics of the reverse biased heterojunction diodes fabricated of *n*-ZnO/p-Si, both with and without HfO_2_. These diodes show properties of the space-charge limited current (SCLC) flow. The existence of multiple charge traps in the intrinsic region of the device is responsible for this phenomenon.

In conclusion, the H2Z5 heterojunction diode exhibits the highest rectification ratio (RR = 6.56 × 10^6^) and barrier height (Φ_b_ = 0.817 eV) together with the lowest values of ideality factor (*n* = 2.124), the reverse leakage current (*I*_rlc_ = 20.2 nA) and interface states (*N*_ss_ = 5.01 × 10^11^ eV^–1^ cm^–2^). Therefore, the adjusting of the ZnO thickness in *n*-ZnO/p-Si heterojunction diodes with HfO_2_ interface layer play a significant role in obtaining the requested electrical properties of the heterojunction diodes.

The obtained results show that a bilayer ZnO/HfO_2_ thin films could be utilized for development of MIS or MOS devices. Our findings add to the ongoing discussion, the H2Z5 sample holds promise for the development of more effective electrical devices, including UV sensors, solar cells, and heterojunctions.

## Supporting Information Available

Detailed illustration of the device fabrication process: preparation of solutions via sol–gel method and the coating procedure using spin coating technique; electrical measurement data: *J*–*V* and *J*^2^–*V* results for SCLC theory of the devices (PDF)
