# Supplementary material for: Electrical Properties of Schottky Devices from HfO2 and ZnO/HfO2 Thin Films: Morphological, Structural, and Optical Investigations
Source: ACS Omega. 2025 Feb 12;10(7):6520–33. doi: 10.1021/acsomega.4c06878 (PMC11866202; doi:10.1021/acsomega.4c06878)
Supplement: Supplementary file 1 — ao4c06878_si_001.pdf [file ao4c06878_si_001.pdf]

Supporting Information

Electrical Properties of Schottky Devices from HfO_2 and ZnO/HfO_2 Thin Films: Morphological, Structural, and Optical Investigations

Ayten SEÇKİN[a] * , Haluk KORALAY[b]

[a] Basic and Engineering Sciences Central Laboratory Application and Research Center (GUTMAM), Gazi University, Ankara, Turkiye

[b]Department of Physics, Faculty of Science, Gazi University, Ankara, Tuail: aytenseckin@gazi.edu.tr

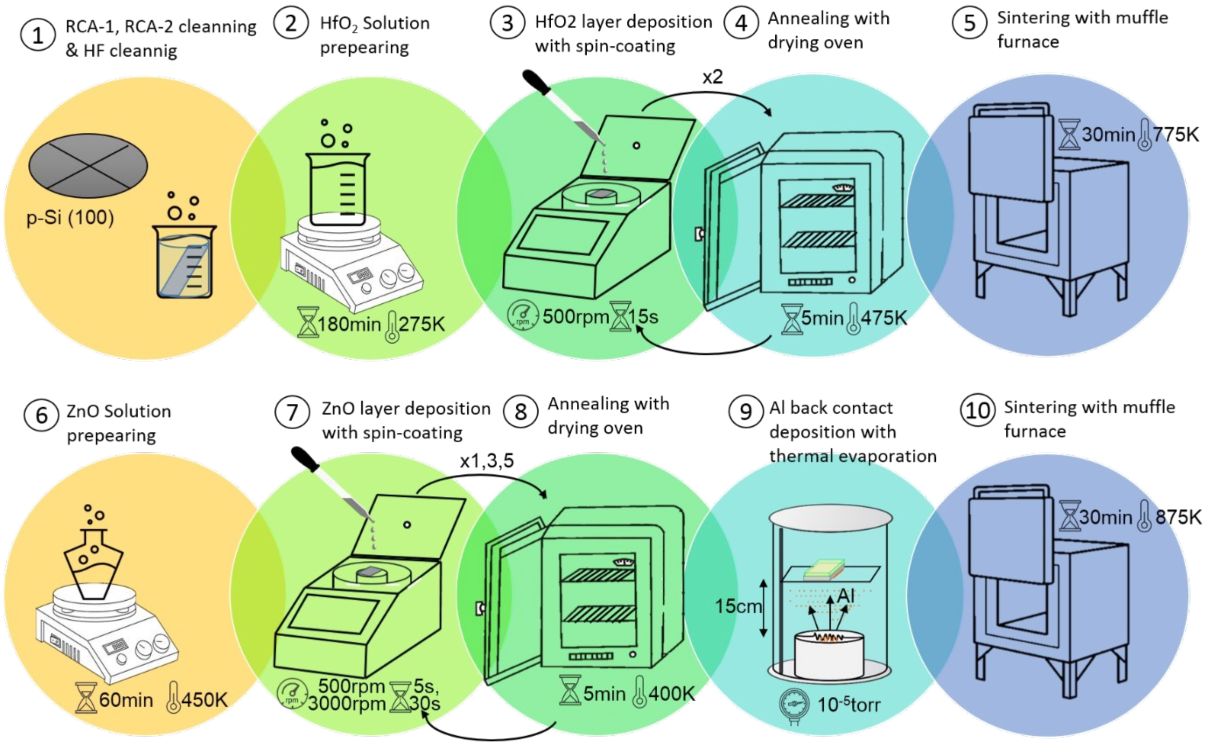

Figure S1. Graphical abstract

(a)

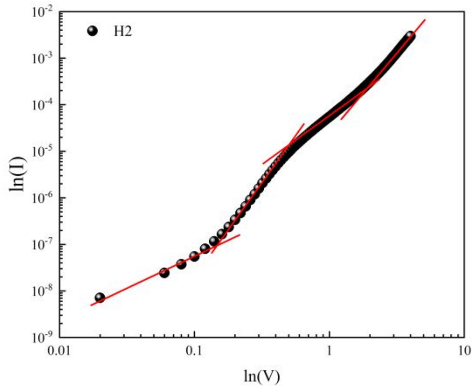

(b)

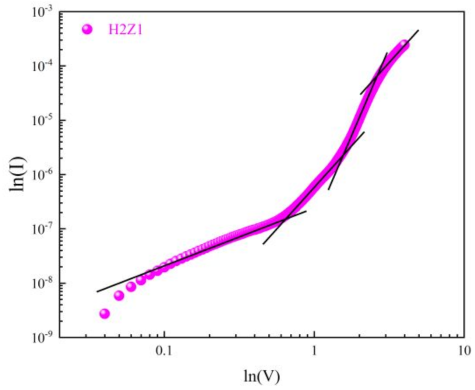

(c)

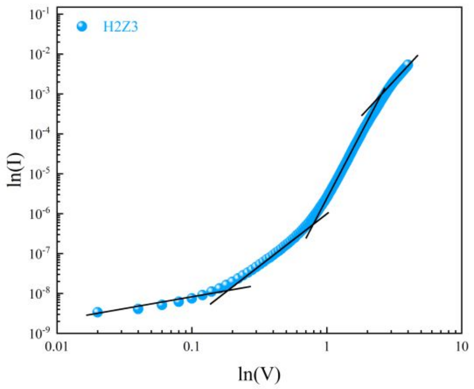

(d)

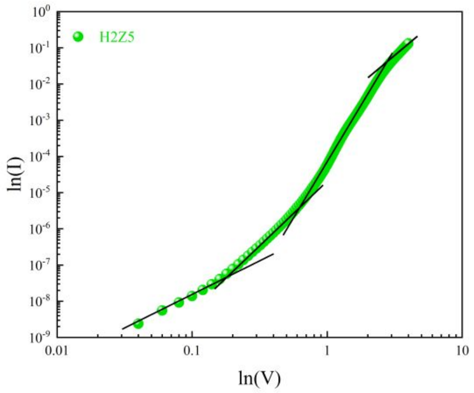

Figure S2. ln(I) versus ln(V) plots of (a)H2, (b)H2Z1, (c)H2Z3, (d)H2Z5

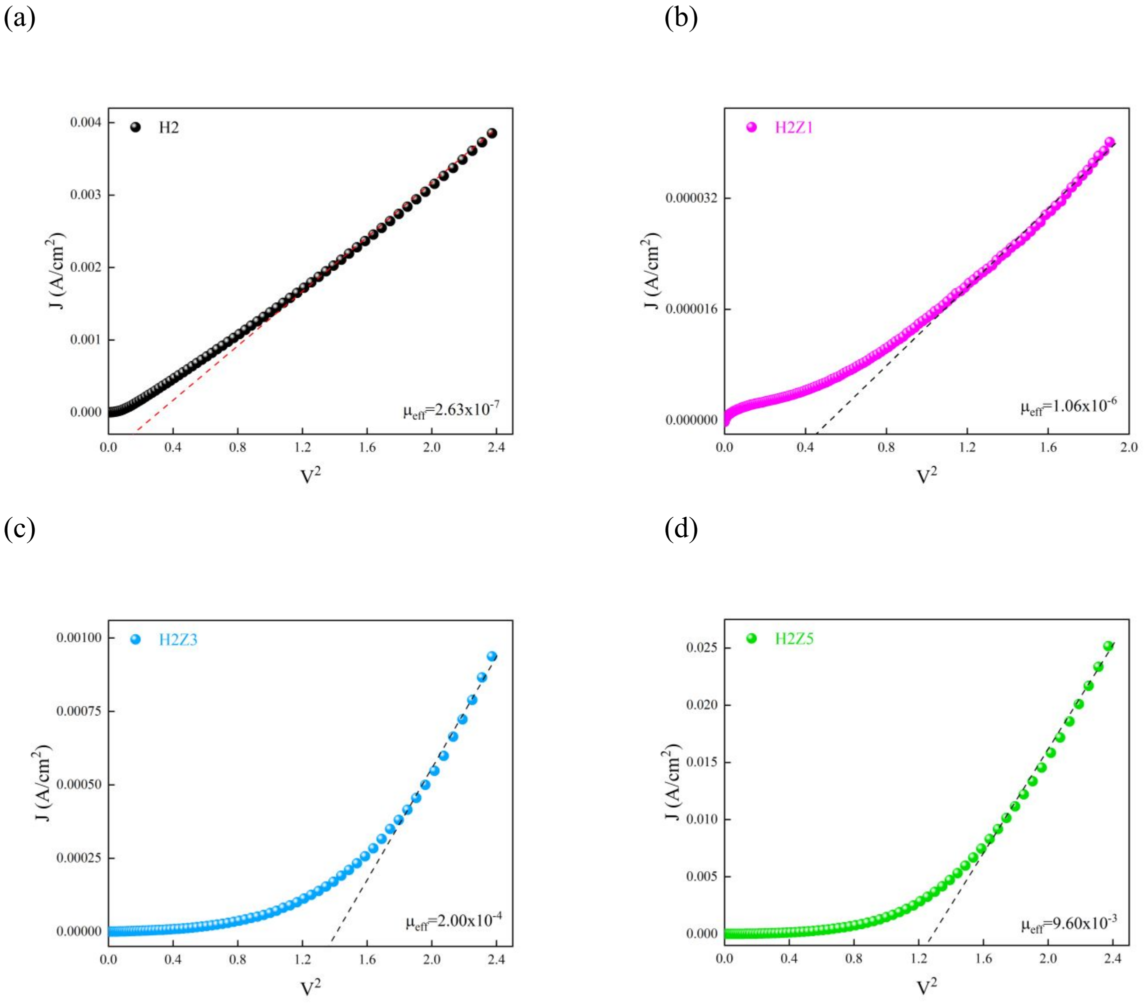

Figure S3. J versus V^2 plots of (a)H2, (b)H2Z1, (c)H2Z3, (d)H2Z5

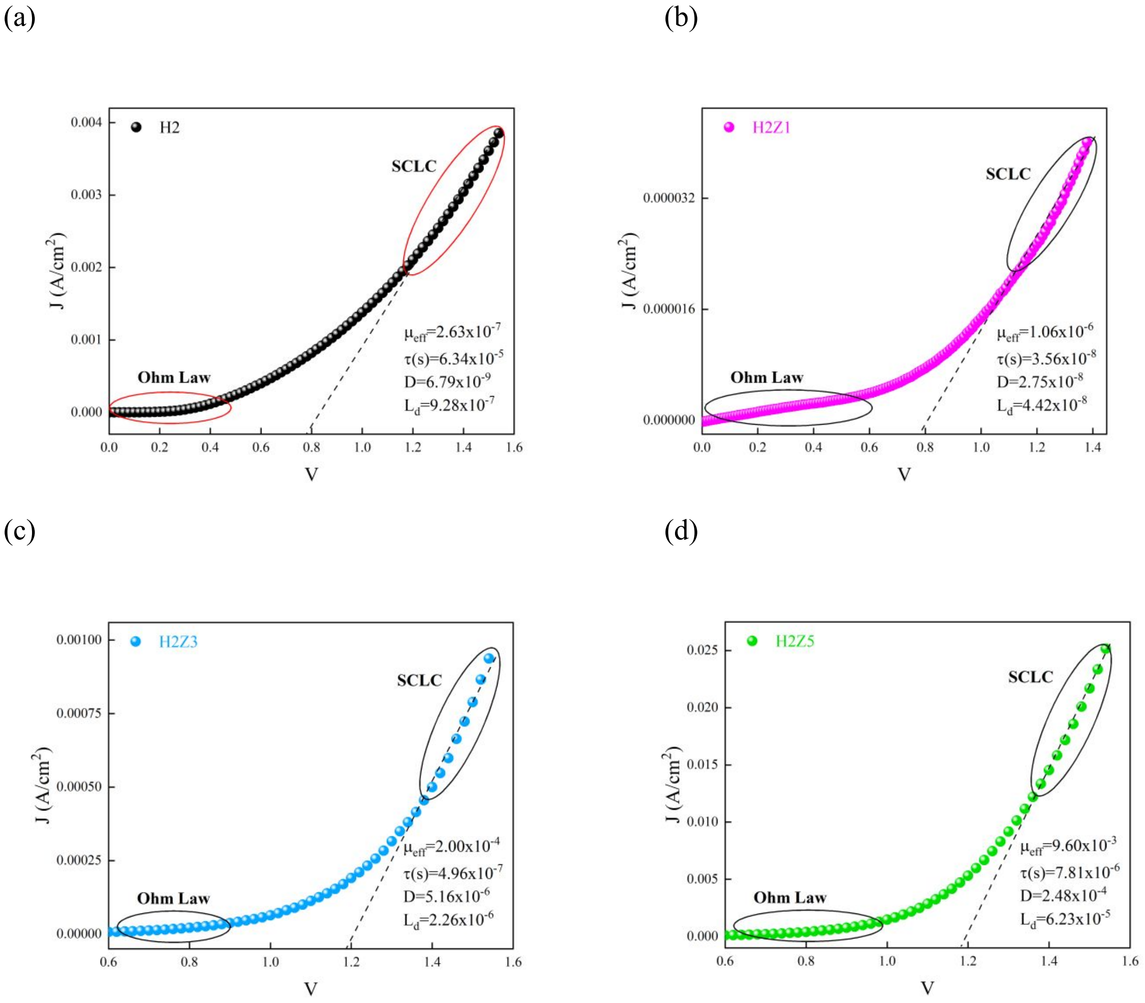

Figure S4. J versus V plots of (a)H2, (b)H2Z1, (c)H2Z3, (d)H2Z5
